# Supplementary material for: Genome-wide CRISPR Screens in T Helper Cells Reveal Pervasive Crosstalk between Activation and Differentiation
Source: Cell. 2019 Feb 7;176(4):882–896.e18. doi: 10.1016/j.cell.2018.11.044 (PMC6370901; doi:10.1016/j.cell.2018.11.044)
Supplement: Data S2. Processed Data from All the Steps of the Analysis, Related to Figure 1 [file mmc2.zip › supplemental data/motif analysis/Bhlhe40_homer/homerResults/motif1.similar.html]

motif1

## Information for motif1

A
T
C
G
A
T
C
G
C
A
T
G
T
A
G
C
C
T
G
A
G
A
T
C
C
A
T
G
A
C
G
T
A
C
T
G
G
T
A
C
  
Reverse Opposite:  

C
A
T
G
T
G
A
C
T
C
G
A
G
T
A
C
C
T
A
G
G
A
C
T
A
T
C
G
G
T
A
C
T
A
G
C
T
A
G
C
  

|  |  |
| --- | --- |
| p-value: | 1e-502 |
| log p-value: | -1.157e+03 |
| Information Content per bp: | 1.652 |
| Number of Target Sequences with motif | 567.0 |
| Percentage of Target Sequences with motif | 47.25% |
| Number of Background Sequences with motif | 1333.6 |
| Percentage of Background Sequences with motif | 3.14% |
| Average Position of motif in Targets | 102.6 +/- 47.7bp |
| Average Position of motif in Background | 97.5 +/- 79.6bp |
| Strand Bias (log2 ratio + to - strand density) | 0.0 |
| Multiplicity (# of sites on avg that occur together) | 1.09 |
| Motif File: | file (matrix) reverse opposite |

### Similar de novo motifs found

|  |  |  |  |  |  |  |  |
| --- | --- | --- | --- | --- | --- | --- | --- |
| Rank | Match Score | Redundant Motif | P-value | log P-value | % of Targets | % of Background | Motif file |
| 1 | 0.946 | T A C G C A G T A G T C C T G A A G T C A C T G A C G T A C T G | 1e-485 | -1118.919004 | 59.67% | 7.15% | motif file (matrix) |
| 2 | 0.987 | G A T C A C T G A G T C C G T A A G T C A C T G A C G T A C T G G T A C T A C G A T C G T A C G | 1e-434 | -1000.940726 | 40.42% | 2.50% | motif file (matrix) |
| 3 | 0.898 | C T A G T C G A G T C A C G T A A G T C A C T G A C G T A C T G G T C A A T G C C A G T A G T C | 1e-406 | -937.088784 | 46.42% | 4.46% | motif file (matrix) |
| 4 | 0.883 | C A G T A T G C C T G A G A T C T C A G G C A T A T C G G T C A A G T C T C A G | 1e-340 | -783.557951 | 59.08% | 11.53% | motif file (matrix) |
| 5 | 0.825 | C G A T G A T C C T A G G T A C C T A G A G C T T A C G A T G C | 1e-268 | -618.606557 | 47.08% | 8.58% | motif file (matrix) |
| 6 | 0.749 | A C T G T C G A C G T A C G T A A G T C A C T G A C G T A C T G G T A C T A G C | 1e-92 | -212.007478 | 12.08% | 1.23% | motif file (matrix) |
| 7 | 0.752 | A G T C C T G A G T A C T C A G A G C T T C A G G T C A A T G C G T C A A G T C | 1e-73 | -169.712363 | 19.00% | 4.55% | motif file (matrix) |
| 8 | 0.743 | G A T C T A C G A G T C A G T C T C G A A G T C T C A G A G C T T A C G T G C A T A G C T G C A | 1e-65 | -151.327934 | 11.00% | 1.59% | motif file (matrix) |
| 9 | 0.653 | A C T G T G A C T G C A A G T C A T C G G C T A C T A G C G T A A T C G T C A G T C A G G A T C | 1e-51 | -118.638165 | 12.08% | 2.58% | motif file (matrix) |
| 10 | 0.784 | C T A G C G T A C G T A A G T C A C T G A C G T A C T G A G T C | 1e-45 | -105.358415 | 6.25% | 0.67% | motif file (matrix) |
| 11 | 0.660 | A G T C C G T A A G T C A C T G A C G T A C G T A C G T A G T C | 1e-43 | -99.445412 | 6.17% | 0.71% | motif file (matrix) |
| 12 | 0.645 | T C A G G C T A C T G A A T G C C A T G A C G T A T C G G C T A A T G C C G A T | 1e-34 | -78.461759 | 3.67% | 0.26% | motif file (matrix) |
| 13 | 0.660 | A G T C A C T G C G A T A C T G A G T C A T G C C G A T C T G A | 1e-24 | -55.928862 | 11.33% | 4.18% | motif file (matrix) |
| 14 | 0.699 | C A T G A T G C A C T G A G T C C T A G G A C T T A C G A G T C T C G A A G C T | 1e-20 | -47.791998 | 15.33% | 7.27% | motif file (matrix) |
| 15 | 0.621 | A G C T A C T G A G T C A C T G A G T C T A C G A T G C T C A G A G C T A C T G G A T C A T C G | 1e-18 | -42.429673 | 14.42% | 7.00% | motif file (matrix) |
| 16 | 0.639 | A C T G A C G T T A G C A T C G G A T C A T C G A C G T A T C G G T C A A C G T G A T C A G T C | 1e-16 | -38.138418 | 1.75% | 0.13% | motif file (matrix) |
